# Semi-quantized Spin Pumping and Spin-Orbit Torques in Topological Dirac Semimetals

**DOI:** 10.1038/s41598-019-55802-7

**Published:** 2019-12-23

**Authors:** Takahiro Misawa, Kentaro Nomura

**Affiliations:** 1 Institute for Solid State Physics, University of Tokyo, 5-1-5 Kashiwanoha, Kashiwa, Chiba 277-8581 Japan; 2 Institute for Materials Research, Tohoku University, Sendai, 980-8577 Japan; 3 Center for Spintronics Research Network, Tohoku University, Sendai, 980-8577 Japan

**Keywords:** Materials science, Condensed-matter physics, Spintronics

## Abstract

We study the time-development processes of spin and charge transport phenomena in a topological Dirac semimetal attached to a ferromagnetic insulator with a precessing magnetization. Compared to conventional normal metals, topological Dirac semimetals manifest a large inverse spin Hall effect when a spin current is pumped from the attached ferromagnetic insulator. It is shown that the induced charge current is semi-quantized, i.e., it depends only on the distance between the two Dirac points in momentum space and hardly depends on the disorder strength when the system remains in the topological Dirac semimetal phase. As an inverse effect, we show that the electric field applied to the topological Dirac semimetal exerts a spin torque on the local magnetization in the ferromagnetic insulator via the exchange interaction and the semi-quantized spin Hall effect. Our study demonstrates that the topological Dirac semimetal offers a less-dissipative platform for spin-charge conversion and spin switching.

## Introduction

Manipulation of magnetization direction by applying electric currents is intended to be used in future magnetic devices, allowing information to be written electrically^1–3^. Spin-orbit torques such as those induced by the spin-Hall effect^4,5^ and the Rashba-Edelstein effect^6,7^ have recently been examined for a variety of materials. However, such current driven magnetization switching suffers from Joule heating problems for device applications. A significant effort has been made in the search for materials having high efficiency^1–3,8^. Obviously, one can expect a large spin-torque effect in systems with a strong spin-orbit interaction^9^. However, theoretical studies on the spin-torque effect in such strongly coupled spin-orbit systems are beyond the scope of the conventional theory^10^.

As an inverse effect of the electrically induced spin-torque effect, a charge current is generated in a metal by the precessing magnetization of an attached ferromagnetic insulator^11^. This phenomenon can be interpreted as a combination of the spin-pumping effect and the inverse spin-Hall effect or the inverse Rashba-Edelstein effect^12,13^. The strength of the spin-Hall effect and the inverse spin-Hall effect is characterized by the spin-Hall angle ${\theta }_{{\rm{SH}}}=\mathrm{(2}e/\hslash ){j}_{s}/{j}_{c}$, where *j*_*c*_ is the charge current generated by an applied electric field and *j*_*s*_ is the spin current induced by the spin-Hall effect. For typical metals such as Pt, Au, and Ta, the value of the spin-Hall angle is ~0.1^8,14^. Materials with larger spin-Hall angles have been researched for application in devices.

Quantum spin Hall insulators^15,16^ have a quantized spin Hall conductivity and a vanishing longitudinal conductivity. In systems with boundaries the spin-momentum locked gapless edge states lie inside the bulk gap. Several theoretical studies have been conducted on coupled spin dynamics and charge transport at the interface between a quantum spin Hall insulator and a ferromagnetic material, such as magnetically generated charge currents^17–24^. However, magnetization reversal in these systems might be difficult because the interface area is small. To exert a large spin torque on the magnetization and reverse its direction, a two-dimensional interface is necessary. Recently, the interface between a three-dimensional (3D) topological insulator and a ferromagnetic material has been realized experimentally. Relatively large spin Hall angles measured using spin transfer torque ferromagnetic resonance, spin-charge conversion, and magnetization reversal have been reported^25–29^. With the successful research on the spintronics phenomena using topological insulators, studies on a wider range of 3D topological materials with higher functionalities are desired.

**Figure 1:**
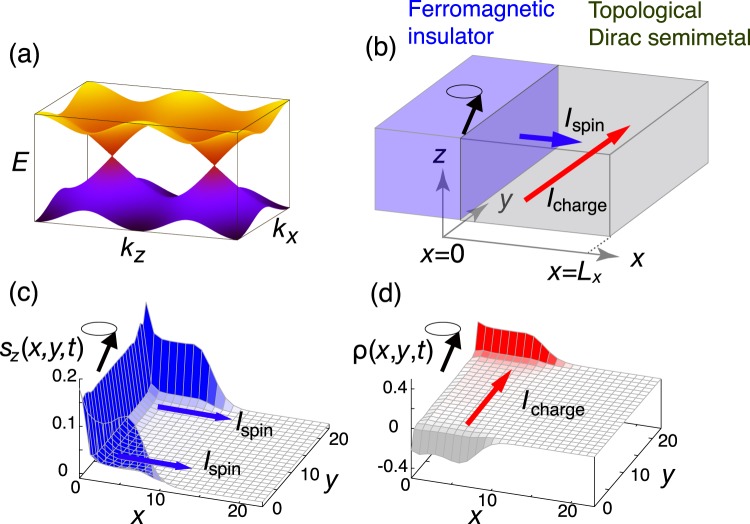
Setup of a topological Dirac semimetal attached to a ferromagnetic insulator and snapshot of charge and spin density. (**a**) TDSM has a couple of Dirac points where the two bands touch at the points which reside on the *k*_*z*_ axis in the momentum space. (**b**) Schematic illustration of the bilayer system of a ferromagnetic insulator and a TDSM. Precessing magnetization causes spin injection and generates transverse charge current. (**c**) Snapshot of the *z* component of spin polarization ${s}_{z}(x,y,t)$ at $t/T=1.0$ for ${L}_{x}={L}_{y}=2{L}_{z}=24$, *J* = 0.5, *T* = 20, *ξ* = 0.1, and *m* = 0. The spin polarization is injected into the TDSM. (**d**) Snapshot of the charge polarization $\rho (x,y,t)$ at *t*/*T* = 1.0. The charge polarization is induced by the injected spins.

In this work, we consider a topological Dirac semimetal (TDSM)^30–37^ as an effective platform for spin-charge conversion and study magnetically induced charge pumping and current-induced magnetization reversal. TDSMs are 3D gapless materials with pair(s) of doubly degenerate Dirac cones, separated in the momentum space along a rotational axis (Fig. 1(a)) and protected by rotational symmetry^31,32^. The degeneracy is attributed to time-reversal and space-inversion symmetries. Na_3_Bi^30,33^ and Cd_3_As_2_ ^34–37^ have been theoretically and experimentally confirmed to be TDSMs. One of the prominent features of this system, which plays an essential role in this work, is that when the Fermi level resides at the Dirac point, the longitudinal conductivity vanishes (i.e., *σ*_*xx*_ = 0)^38,39^ while the spin Hall conductivity is semi-quantized in the bulk limit (i.e., ${\sigma }_{xy}^{z}=\frac{e}{4{\pi }^{2}}\Delta k$, where Δ*k* is the distance between Dirac points)^40^, indicating that the spin Hall angle *θ*_SH_ diverges in the bulk. The large spin Hall angle causes the strong spin-torque effect induced by the small longitudinal charge current. Another important point is that helical surface modes emerge at the boundary of a TDSM in which the spin-up electrons go one way while the spin-down electrons go the opposite way. Under an applied electric field, a charge current flows with transverse spin polarization similar to the Rashba-Edelstein effect. Naively, one can expect that the spin polarization generated in the helical modes in TDSMs is larger than that in the Rashba systems and the surface of topological insulators since only *z* component of spin is approximately conserved.

Using the numerical time-development formalism of quantum systems^41,42^, we study pumped charge currents by precessing magnetization and spin-orbit torque. This formalism enables us to analyze the transport phenomena and spin-torque effects in the heterostructures beyond conventional theories such as the linear response theory. We claim that the Dirac electrons exert spin-orbit torques on the local magnetization of the ferromagnet at the interface, even when the Fermi level resides at the Dirac point. As in conventional bilayer systems of a ferromagnet and a metal, anti-dumping torques are induced by the bulk spin Hall current and a field-like torque is induced by the accumulated electron spins at the interface.

## Results

### Model and methods

TDSMs are characterized by a pair of Dirac points along the *k*_*z*_ axis, stabilized by discrete rotational symmetry (see Fig. 1(a)). To describe the electronic states in a TDSM on a simple lattice system, we employ the effective tight-binding Hamiltonian

1 $${ {\mathcal H} }_{{\rm{TDSM}}}({\boldsymbol{k}})=\,\sin \,{k}_{x}{\alpha }_{3}+\,\sin \,{k}_{y}{\alpha }_{4}+(2+m-\,\cos \,{k}_{x}-\,\cos \,{k}_{y}-\,\cos \,{k}_{z}){\alpha }_{5},$$

which is connected, in the long wavelength limit, to the four-band $k\cdot p$ Hamiltonian derived for Na_3_Bi^34^, where

2 $${\alpha }_{3}=(\begin{array}{cc}0 & {\sigma }_{z}\\ {\sigma }_{z} & 0\end{array}),{\alpha }_{4}=(\begin{array}{cc}0 & -{\rm{i}}\\ {\rm{i}} & 0\end{array}),{\alpha }_{5}=(\begin{array}{cc}I & 0\\ 0 & -I\end{array})$$

are 4 × 4 matrices, *σ*_*i*_ (*i* = *x*, *y*, *z*) being the Pauli matrices acting on the spin indices. The Dirac points are located at ${{\boldsymbol{k}}}_{{\rm{D}}}=(0,0,\pm {\cos }^{-1}(m))$ for |*m*| < 1. We consider a TDSM attached to a ferromagnetic insulator at *x* = 0 plane (see Fig. 1(b)), whose Hamiltonian is given by $H(t)={H}_{{\rm{T}}{\rm{D}}{\rm{S}}{\rm{M}}}+{H}_{{\rm{e}}{\rm{x}}{\rm{c}}}(t)$, where

3 $${H}_{{\rm{TDSM}}}=\sum _{j,\mu =x,y,z}\,({c}_{j+{{\boldsymbol{e}}}_{\mu }}^{\dagger }{T}_{\mu }{c}_{j}+{\rm{H}}.{\rm{c}}.)+(2+m)\sum _{j}\,{c}_{j}^{\dagger }{\alpha }_{5}{c}_{j}$$

is the real space representation in the second quantization formalism. In Eq. (3), ${c}_{j}^{\dagger }({c}_{j})$ represents the four-component fermion creation (annihilation) operator defined on a site *j* on the 3D cubic lattice spanned by three orthogonal unit vectors ${{\boldsymbol{e}}}_{\mu }=x,y,z$. The matrix is defined as ${T}_{x}=(-\,{\alpha }_{5}+i{\alpha }_{3})/2$, ${T}_{y}=(\,-\,{\alpha }_{5}+i{\alpha }_{4})/2$, ${T}_{z}={\alpha }_{5}/2$. On the other hand,

4 $${H}_{{\rm{exc}}}(t)=\sum _{j}\,J\hat{{\boldsymbol{M}}}(t)\cdot ({J}_{0}(x){c}_{j}^{\dagger }{\boldsymbol{s}}{c}_{j}+{J}_{3}(x){c}_{j}^{\dagger }({\boldsymbol{s}}{\alpha }_{5}){c}_{j}),$$

describes the exchange interaction at the interface between the TDSM and the ferromagnet. $\hat{{\boldsymbol{M}}}$ is the unit vector in the direction of local magnetization in the ferromagnetic insulator, ${J}_{0}(x)={J}_{0}\times \exp (-\,x/\xi )$ and ${J}_{3}(x)={J}_{3}\times \exp (-\,x/\xi )$ are the coupling constants of the exchange interactions with the penetration length *ξ*, ${\boldsymbol{s}}={\rm{diag}}({\boldsymbol{\sigma }},{\boldsymbol{\sigma }})$ is the spin operator of the electrons in the TDSM. For simplicity, we take ${J}_{0}={J}_{3}=J$ in this study. We perform the calculation for a 3D system whose size is given by ${L}_{x}\times {L}_{y}\times {L}_{z}$. In this paper, we only treat the TDSM protected by the spin Chern number. We note that a different type of the TDSM protected by the mirror Chern number is proposed^43^.

First, we consider the spin and charge pumping due to a precessing magnetization. The ferromagnetic insulator is assumed to be excited by microwave radiation (as done in the experiment^11^) resulting in a steady precession about the effective field close to the ferromagnetic resonance condition:

5 $$\hat{{\boldsymbol{M}}}(t)=(\sqrt{1-{M}_{0}^{2}}\,\cos (\omega t),\sqrt{1-{M}_{0}^{2}}\,\sin (\omega t),{M}_{0}),$$

in Eq. (5), where $\omega =2\pi /T$ is the frequency with *T* being the period of the precession. In the following analysis, we set *T* = 20 and *M*_0_ = 0. When the magnetization begins to precess, an electronic state of the TDSM evolves to a non-equilibrium state $\Psi (t)$. $\Psi (t)$ is obtained by solving the time-dependent Shrödinger equation $i(\partial /\partial t)|\Psi (t)\rangle =H(t)|\Psi (t)\rangle$ numerically with a time step of Δ*t*. The time development of the wave function is given by $\Psi (t+\Delta t)=U(t+\Delta t,t)\Psi (t)$, where *U*(*t* + Δ*t*, *U*(*t*) is the unitary time-evolution operator defined as

6 $$U(t+\varDelta t,t)={\mathscr T}\,\exp [\,-\,{\rm{i}}{\int }_{t}^{t+\Delta t}dt^{\prime} H(t^{\prime} )].$$

Here, ${\mathscr T}$ is the time-ordering operator. Using the formula given in literature^41,42^, we decompose *U*(*t* + Δ*t*, *t*) into a product of small exponential operators and perform real-time evolution as matrix-vector multiplication (for details, see the Methods section). In this method, the diagonalization of the Hamiltonian is necessary only for preparing the initial wave function and the numerical cost is significantly reduced. In the following analysis, we impose a periodic boundary condition in the *z* direction and a fixed boundary condition in the *x* direction. Depending on the quantities to be computed, the periodic boundary condition or the fixed boundary condition is implemented in the *y* direction. In the following, we set the Fermi energy at the Dirac point.

### Spin density and charge density

To characterize the time evolution of the spin and charge densities in the TDSM, we introduce the quantity ${n}_{\sigma }(x,y,z{)|}_{t}=\langle \Psi (t)|{c}_{j\sigma }^{\dagger }{c}_{j\sigma }|\Psi (t)\rangle$ as the density of electrons with spin $\sigma =\uparrow ,\downarrow$ at time *t* and position $j=(x,y,z)$. The precession motion of the magnetization starts at *t* = 0. The subsequent time development of the electrons is given by

7 $${N}_{\sigma }(x,y,t)\equiv \frac{1}{{L}_{z}}\sum _{z}\,[{n}_{\sigma }(x,y,z{)|}_{t}-{n}_{\sigma }(x,y,z{)|}_{t=0}\mathrm{]}.$$

Since we are interested in the spin and charge propagating in the *x* and/or *y* directions, the unimportant variable *z* is integrated. For this purpose, we terminate the system by applying the fixed boundary condition in the *x* and *y* directions, while the periodic boundary condition is implemented in the *z* direction. The time evolution of the charge and spin densities is given by

$$\begin{array}{rcl}\rho (x,y,t) & = & -e({N}_{\uparrow }(x,y,t)+{N}_{\downarrow }(x,y,t))\\ {s}_{z}(x,y,t) & = & \frac{1}{2}({N}_{\uparrow }(x,y,t)-{N}_{\downarrow }(x,y,t)).\end{array}$$

In Fig. 1(c,d), we present the snapshots of $\rho (x,y,t)$ and ${s}_{z}(x,y,t)$ at $t/T=1$. Immediately after the magnetization begins to precess, spin polarization of the Dirac electrons is generated near the interface at *x* = 0. The accumulated spins then begin to propagate into the TDSM. As the TDSM possesses the gapless surface states while the density of states vanishes in the bulk, the spins propagate mainly on the surface ($y=0$ and $y={L}_{y}-1$) as shown in Fig. 1(c). In response to the spin propagation, the electrons are pumped in the *y* direction as shown in Fig. 1(d). This development of the charge polarization is related to the charge current flowing in the *y* direction, anticipated from from the inverse spin Hall effect and the inverse Rashba-Edelstein effect^13,44^.

### Charge current

Next, we directly compute the charge current induced by the precessing magnetization. For this purpose, we apply the periodic boundary condition in the *y* direction (the direction in which the current flows), in addition to the *z* direction. The charge current operator is given by

8 $${I}_{c}^{y}=-\,{\rm{i}}\,\sum _{j}\,({c}_{j+{{\boldsymbol{e}}}_{y}}^{\dagger }{T}_{y}{c}_{j}-{c}_{j}^{\dagger }{T}_{y}^{\dagger }{c}_{j+{{\boldsymbol{e}}}_{y}})\mathrm{}.$$

**Figure 2:**
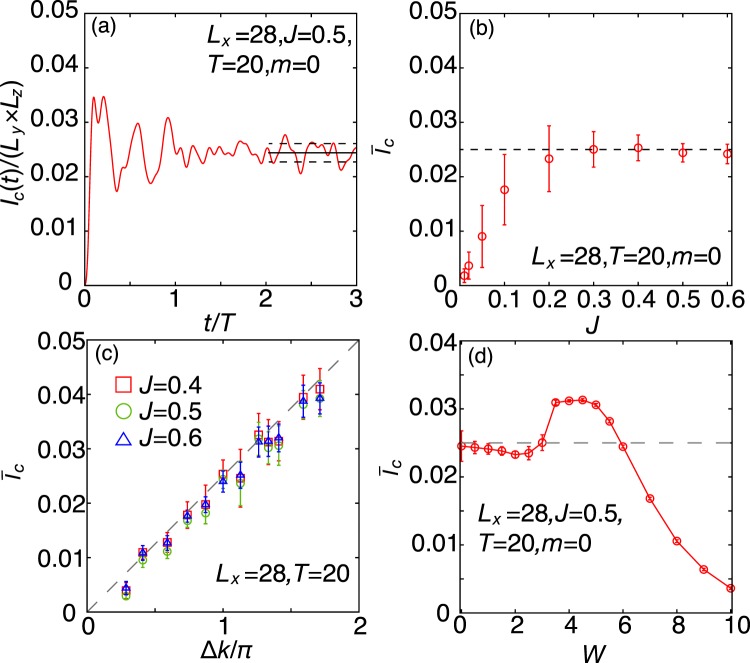
Charge current as a function of time, exchange coupling, distance between Dirac points, and disorder strength. (**a**) Time dependence of the charge current for the case $J=0.5$, $T=20$, $m=0$, $\xi =0.1$, and ${L}_{x}=2{L}_{y}=2{L}_{z}=28$. We regard the width of the oscillation (standard deviation) of the charge current in $3\ge t/T\ge 2$ as the error bars (shown in broken lines). (**b**) Averaged charge current (${\bar{I}}_{c}$) as a function of the exchange coupling *J*. (**c**) Averaged charge current (${\bar{I}}_{c}$) as a function of the distance between the Dirac points for $J=0.4,0.5$, and 0.6 (${L}_{x}=28$). (**d**) Averaged charge current (${\bar{I}}_{c}$) as a function of the disorder strength *W*.

Its expectation value $\langle \Psi (t)|{I}_{c}^{y}|\Psi (t)\rangle$ is plotted as a function of time in Fig. 2(a). After the magnetization begins to precess, the charge current increases at the initial stage of the time evolution. For $t\ge 2T$, the charge current converges to a constant value with some oscillations. We obtain the average value of the charge current from the relation:

9 $${\bar{I}}_{c}=\frac{1}{\Delta t}{\int }_{{t}_{0}}^{{t}_{0}+\Delta t}\,dt\langle \Psi (t)|{I}_{c}^{y}|\Psi (t)\rangle $$

We consider ${t}_{0}=2T$ and $\Delta t=T$. Figure 2(b) shows the *J*-dependence of (${\bar{I}}_{c}$); the figure clearly indicates that (${\bar{I}}_{c}$) increases with *J* from zero and converges to a constant value when *J* is large enough. We found that the average value of the current is given by

10 $${\bar{I}}_{c}=\nu \frac{e}{2\pi }\frac{\partial \phi }{\partial t},$$

where $\nu ={L}_{z}\Delta k\mathrm{/2}\pi$ is the number of the helical channels at the surface and $\phi ={\tan }^{-1}({M}_{y}/{M}_{x})$. This is examined by studying the dependence of the distance between the Dirac points in momentum space, $\Delta k=\mathrm{2|}{{\boldsymbol{k}}}_{{\rm{D}}}|=2{\cos }^{-1}(m)$, on the charge current by tuning the parameter *m*. By increasing Δ*k*, we observe that the pumped charge current increases linearly with Δ*k* as shown in Fig. 2(c). This result indicates that the charge current is governed by Fermi arcs in the TDSM since the Fermi arcs increase in length as Δ*k* increases.

### Effects of disorder

We consider the effects of disorder by introducing site-dependent random potentials

$$V=\sum _{i}\,{\varepsilon }_{i}{c}_{i}^{\dagger }{c}_{i},$$

where ${\varepsilon }_{i}\in [-\,W/2:W/2]$ is random number and *W* characterizes the strength of disorder. Figure 2(d) shows the disorder dependence of the induced charge current. We take 72 independent realizations for performing the disorder average. We calculate both the standard errors of the current with respect to the disorders and the disorder-average oscillations of the current, and regard the larger one as the error bars. At weak disorder ($0\lesssim W\lesssim 3$), the charge current is insensitive to the disorder strength and approximately takes the semi-quantized value. At strong disorder ($3\lesssim {\rm{W}}$) where the TDSM phase is broken by disorder, the time-averaged value of the induced charge current deviates from the semi-quantized value: it first increases and then decreases as *W* increases. These results indicate that the magnetically induced charge current is robust against disorder when the system is in the TDSM phase. The increase in charge current in the strong disorder regime could be explained as follows. It is known that the density of states and the bulk conductivity at the Dirac point remains vanishing in the Dirac semimetal phase^38^. When the disorder strength exceeds the critical value, the system turns into the diffusive metallic phase where the density of states and the bulk conductivity become finite. The increased density of states could enhance the bulk contribution to the magnetically induced charge current. We note that the similar disorder induced phase transition occurs for the Weyl semimetals^45,46^.

### Magnetization switching and spin torque

From the above discussion, one can infer that the magnetization dynamics in the ferromagnetic insulator generates the charge current in the TDSM. In the rest of this work, we study the spin-torque effect and dynamics of the magnetization induced by the electric field in the TDSM. Magnetization dynamics in the ferromagnetic insulator are described by the phenomenological Landau-Lifshitz-Gilbert (LLG) equation:

11 $$\frac{d\hat{{\boldsymbol{M}}}}{dt}=-\gamma \hat{{\boldsymbol{M}}}\times {{\boldsymbol{H}}}_{{\rm{eff}}}+\alpha \hat{{\boldsymbol{M}}}\times \frac{d\hat{{\boldsymbol{M}}}}{dt}+\frac{1}{\hslash }\hat{{\boldsymbol{M}}}\times (\sum _{x}\,{J}_{0}(x){\langle {\boldsymbol{s}}\rangle }_{x}+{J}_{3}(x){\langle {\boldsymbol{s}}{\alpha }_{5}\rangle }_{x})$$

**Figure 3:**
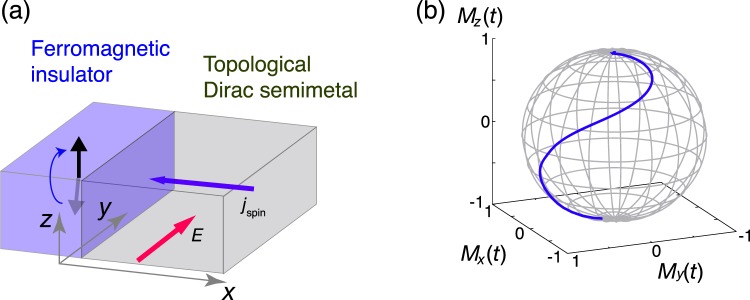
Set up of spin switching and typical trajectory of magnetization. (**a**) Schematic illustration for magnetization switching. The electric field is applied in the *y* direction and the spin current flows in the *x* direction. (**b**) Typical dynamical behavior of the local magnetization caused by the spin torque at the interface. At *t* = 0, an electric field is switched on. The induced spin current exerts a spin torque on the local magnetization in the ferromagnetic insulator.

Here, we assume that the magnetic insulator is thin enough and the magnetization is uniform in space. In Eq.(11), *γ* is the gyromagnetic ratio, *α* is the Gilbert damping constant, ${\langle {\boldsymbol{s}}\rangle }_{x}$ is the spin density of itinerant Dirac electrons at *x*, and ${{\boldsymbol{H}}}_{{\rm{eff}}}={K}_{z}({M}_{z}{{\boldsymbol{e}}}_{z})$ is the effective magnetic field, which induces the easy-axis anisotropy. The spin density of the Dirac electrons at time *t* and position $j=(x,y,z)$ is given by ${\boldsymbol{s}}(x,y,z{)|}_{t}={\sum }_{\sigma ,\sigma ^{\prime} }\langle \Psi (t)|{c}_{\sigma j}^{\dagger }{{\boldsymbol{\sigma }}}_{\sigma \sigma ^{\prime} }{c}_{\sigma ^{\prime} j}|\Psi (t)\rangle$. The averaged spin density at *x* corresponds to ${\langle {\boldsymbol{s}}\rangle }_{x,t}=\mathrm{(1/}A){\sum }_{y,z}\,{\boldsymbol{s}}(x,y,z{)|}_{t}$, *A* being the area of the interface. The time evolution of $|\Psi (t)\rangle$ and $\hat{{\boldsymbol{M}}}$ is obtained by simultaneously solving the time-dependent Schrödinger equation and the LLG equation. A typical dynamical behavior of the magnetization is shown in Fig. 3, where an electric field is turned on at *t* = 0 via the Peierls substitution of a time-dependent vector potential. Here, we take *γ* = −1, *α* = 0.1, *J* = 0.5, ${L}_{x}=4{L}_{y}=4{L}_{z}=32$, *W* = 0.5, *K*_*z*_ = −0.01, $\xi =0.1$ and ${E}_{y}=\frac{2\pi }{{L}_{y}T}{\Phi }_{0}$, ${\Phi }_{0}$ being the flux quantum. We take 50 independent realizations for performing the disorder average. The direction of the magnetization, originally oriented in −*z* direction (${\bf{M}}=(0,(1-{M}_{0}^{2}{)}^{\mathrm{1/2}},{M}_{0}),{M}_{0}=0.99$) starts to change due to the electrically induced spin torque. After a sufficient passage of time, the magnetization is reversed to +*z* direction.

Due to the spin Hall effect, when an electric field is applied in the *y* direction, a spin current *I*_*z*_ is generated and flows in the *x* direction (Fig. 3(a)). At the interface *x* = 0, the finite spin current is transferred to a spin torque acting on the local magnetization in the ferromagnet. When the spin-orbit interaction at the interface is negligible, this spin-Hall-current induced torque is expressed as the damping-like (DL) torque, ${{\boldsymbol{T}}}_{{\rm{DL}}}\propto {I}_{{\rm{spin}}}^{z}\hat{{\boldsymbol{M}}}\times (\hat{{\boldsymbol{z}}}\times \hat{{\boldsymbol{M}}})$. On the other hand, the contribution of the nonequilibrium spin density at the helical surface state of the TDSM^25^ to the torque is known as the field-like (FL) torque, ${{\boldsymbol{T}}}_{{\rm{FL}}}\propto {S}_{{\rm{edge}}}^{z}\hat{{\boldsymbol{z}}}\times \hat{{\boldsymbol{M}}}$, where ${S}_{{\rm{edge}}}^{z}$ is the accumulated spin density at the interface. Figure 3(b) indicates that the damping-like torque and the field-like torque have the same order of magnitude because both *M*_*x*_, *M*_*y*_ and *M*_*z*_ become finite at the initial stage of the magnetization switching.

## Discussion

In this work, we studied dynamically injected spins into the TDSM from the ferromagnetic insulator with precessing magnetization. The spins propagate and generate the transverse charge current in the TDSM. The response is semi-quantized in the sense that the charge current depends only on the distance between two Dirac points in the momentum space and is robust against disorder when the system remains in the TDSM phase. Moreover, we have studied also its inverse response: when an electric field is applied to the TDSM, a spin torque is exerted on the local magnetization in the ferromagnetic insulator and the direction is reverted. The induced damping-like torque and field-like torque are in same order of magnitude. We note that the magnitudes of the spin Hall conductivity ${\sigma }_{xy}^{z}$ for Na_3_Bi^30^ and Cd_3_As_2_^34^ are comparable with those of conventional metals such as *β*-Ta^8,47,48^. Since the density of states at the Dirac point is vanishingly small, we expect a large spin torque for the TDSM, while the Joule heating accompanying the generation of spin torque is extremely lower than that in conventional metals^47^.

## Methods

To analyze the quantum transport phenomena using the tight-binding model of the topological Dirac semimetals defined in Eqs. (3) and (4), we perform the real-time evolution of the wavefunctions. Here, we present the outline of the method. First, we decompose the Hamiltonian into those for the odd sites and the even sites for $\nu =x,y,z$ directions as follows:

$${H}_{{\rm{TDSM}}}={H}_{\nu ,e}+{H}_{\nu ,o},$$

where ${H}_{\nu ,e}$ (${H}_{\nu ,o}$) denotes the hopping process in which the origin is the even (odd) site. Using the fourth-order Suzuki-Trotter decomposition^41,42^, we decompose the time-evolution operator *U* as follows:

12 $$\begin{array}{ccc}U(t+\Delta t,t) & = & S(\,-\,{\rm{i}}\Delta tp,t+(1-p/2)\Delta t)\\  &  & \times \,S(\,-\,{\rm{i}}\Delta t(1-2p),t+\Delta t/2)\\  &  & \times \,S(\,-\,{\rm{i}}\Delta tp,t+p\Delta t/2)+O(\Delta {t}^{5}),\end{array}$$

where $p={(2-{2}^{1/3})}^{-1}$. Here, *S* is defined as

$$\begin{array}{rcl}S(\eta ,t) & = & {S}_{0}(\eta ,t){e}^{\eta {H}_{{\rm{diag}}}}{S}_{1}(\eta ,t),\\ {S}_{0}(\eta ,t) & = & {e}^{\eta {H}_{x,e}\mathrm{/2}}{e}^{\eta {H}_{x,o}\mathrm{/2}}{e}^{\eta {H}_{y,e}(t\mathrm{)/2}}\\  &  & \times {e}^{\eta {H}_{y,o}(t\mathrm{)/2}}{e}^{\eta {H}_{z,e}\mathrm{/2}}{e}^{\eta {H}_{z,o}\mathrm{/2}}\\ {S}_{1}(\eta ,t) & = & {e}^{\eta {H}_{z,o}\mathrm{/2}}{e}^{\eta {H}_{z,e}\mathrm{/2}}{e}^{\eta {H}_{y,o}(t\mathrm{)/2}}\\  &  & \times {e}^{\eta {H}_{y,e}(t\mathrm{)/2}}{e}^{\eta {H}_{x,o}\mathrm{/2}}{e}^{\eta {H}_{x,e}\mathrm{/2}},\end{array}$$

where *H*_diag_ denotes the diagonal part of the Hamiltonian such as the chemical potential. Using Eq.(12), we perform the real-time evolution of the wave function.
